# Examination under anesthesia in anterior posterior compression pelvic ring injuries; additional assessment of the posterior pelvic ring reveals occult sacroiliac joint instability

**DOI:** 10.1007/s00068-025-03069-1

**Published:** 2026-02-09

**Authors:** Camryn C. Therrien, Kaj ten Duis, Hester Banierink, Jean-Paul P. M. de Vries, Inge H. F. Reininga, Frank F. A. IJpma

**Affiliations:** 1 Department of Trauma Surgery, University of Groningen, University Medical Center Groningen, Groningen, Netherlands; 2 Department of Surgery, University of Groningen, University Medical Center Groningen, Groningen, Netherlands

**Keywords:** Anterior-posterior compression injury, Examination under anaesthesia, Pelvic stability

## Abstract

**Purpose:**

To investigate the added value of directly observing sacroiliac (SI) diastasis with a modified examination under anaesthesia (EUA) protocol, including obturator inlet SI joint projections, in patients with anterior-posterior compression (APC) pelvic ring injuries.

**Methods:**

This prospective cohort study, conducted in a level-1 trauma centre in 2017–2024, included patients > 18 years old with an APC injury, who underwent the modified EUA protocol. The modified protocol includes anterior-posterior projections of the anterior pelvis (as in the original EUA protocol), with additional obturator inlet projections of the SI joints. The primary outcomes were reclassification and detection of occult SI joint diastasis, either anterior or complete, following the modified EUA protocol.

**Results:**

Thirty-five patients, with a mean age of 53, were included, 80% sustaining high-energy trauma. Following modified EUA, 23 (65.7%) of injuries were reclassified compared to classifications from CTs: 9 of 12 (75%) APC1s to APC2, 1 of 12 (8.3%) APC1s to APC3, and 13 of 23 (56.5%) APC2s to APC3. In 16 cases (45.7%), examining the SI joint during EUA enabled localisation of SI diastasis. Eleven patients of 13 (84.6%) with symphyseal diastasis less than the standard cut-off value of 2.5 cm had occult SI diastasis visible on the obturator inlet images. Four of 14 (28.5%) patients with rotational instability less than the standard cut-off value of 1.0 cm had complete SI diastasis visible on obturator inlet images.

**Conclusions:**

A modified EUA protocol, including obturator inlet projections of the SI joints, in patients with APC pelvic ring injuries has been proposed. This protocol allows for direct detection of SI joint diastasis that was not visible on the CT scans, allows for localization of SI joint diastasis, and reveals SI joint diastasis that would remain undetected if only the anterior ring was visualized.

## Introduction

**Fig. 1:**
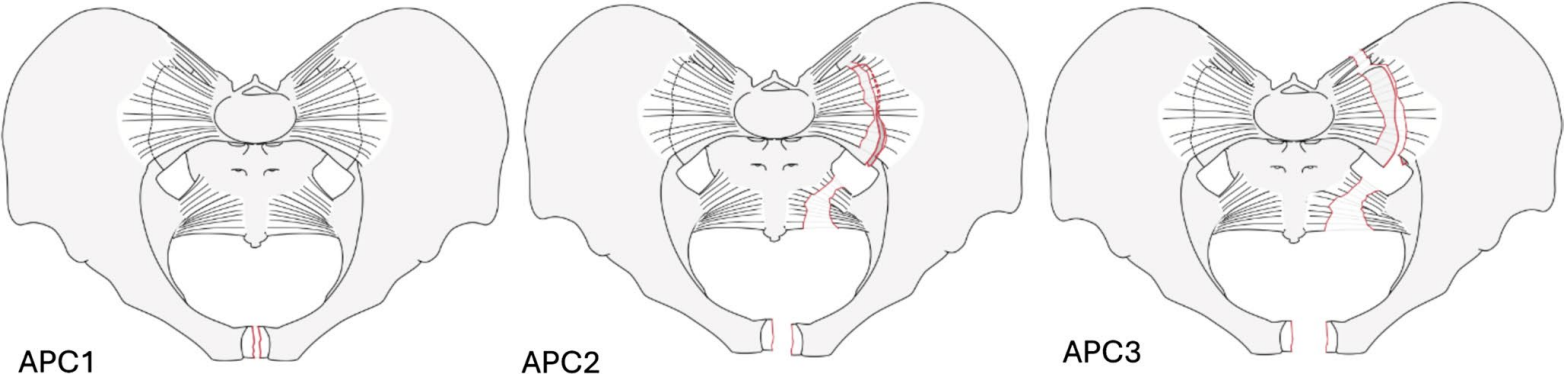
APC1, APC2 and APC3 pelvic ring injuries (1)

Pelvic ring injuries can be classified according to the vector of force inflicted during trauma [1–4]. Anterior posterior compression (APC) injuries, commonly referred to as “open book” injuries, are caused by external rotation forces on the pelvis. This frequently results in fractures of the pubic ramus, along with disruptions in the ligaments of the symphysis, the sacrospinous and sacrotuberous ligaments, and the anterior and posterior sacroiliac (SI) ligaments [4]. According to Young & Burgess, APC injuries are classified into three categories (Fig. 1) based on the structures affected and the amount of instability: APC 1 is a disruption in the anterior pelvic ring, with all SI ligaments intact, APC2 involves additional disruption of the anterior SI ligaments, and APC3 is classified by disruptions of both the anterior and posterior SI ligaments, meaning that the hemipelvis is entirely unstable [4, 5].

Typically, pelvic ring injuries are classified using static radiographic images and CT scans [4]; however, these methods may lead to misclassification of injuries and fail to detect instability [6–9]. The patient’s positioning and the use of a pelvic binder during CTs may limit the detection of diastasis on static radiographs [10, 11]. This can lead to an underestimation of the amount of instability, potentially resulting in inappropriate treatment and poor patient outcomes [6, 7, 12–15]. To address this, examination and fluoroscopy under anaesthesia (EUA) can be utilized to assess pelvic ring stability, aiding in the correct classification of the injury and informing treatment decisions [6, 7, 16].

A symphyseal diastasis of *≥* 2.5 cm during EUA is a commonly used cut-off value in differentiating an APC1 from an APC2 injury [1, 3, 6, 7, 17]. The 2.5 cm cut-off value originated in 1980 from biomechanical studies and is considered the maximum amount of symphyseal diastasis that can occur before the integrity of the SI joints is compromised [3]. This cut-off value is of clinical significance because, according to Sagi et al. [7], patients with a symphyseal diastasis of < 2.5 cm during EUA are assumed to have a stable sacroiliac joint and do not require operative treatment, whereas those with a measurement > 2.5 cm are deemed unstable and require surgical fixation. However, no studies have confirmed this cut-off value. A more recent biomechanical study that attempted to validate the 2.5 cm cut-off value, was unable to confirm it as a reliable differentiation point between APC1 and APC2 injuries [17]. This may be attributed to the considerable morphological variation that can exist in pelvic anatomy [17, 18].

Existing research applying EUA to APC injuries focuses only on the theoretical posterior ring instability by measuring the anterior pelvic ring displacement, using the cut-off values derived from biomechanical studies [6, 7]. However, this EUA approach – focusing solely on the displacement of the anterior pelvic ring – may not account for the complexities and variations in the anatomy of the posterior pelvic ring. Furthermore, this anterior-only EUA approach may fail to localise the SI joint diastasis. Although occult diastasis of the SI joint may be assumed by measuring the amount of displacement in the anterior pelvic ring during EUA, it does not always clarify whether the diastasis is located in the left, right or both SI joints. Therefore, we hypothesised that directly observing the SI joint using obturator inlet projections during EUA is essential to confirm the presence, severity (i.e., partial or complete), and location of SI joint diastasis. To our knowledge, no study has yet directly observed SI joint stability during EUA on patients with APC pelvic injuries.

Therefore, the research questions in this study were: To what extent does observing the SI joints using obturator inlet projections during EUA result in reclassification compared to only using static imaging?; and 2) To what extent does the modified EUA protocol identify occult SI joint diastasis that is not detected on static imaging or by conventional EUA focused only on anterior pelvic displacement?

## Methods

### Patients

This prospective observational cohort study included patients with pelvic ring injuries at a level-1 trauma centre between 2017 and 2024 [19]. The inclusion criteria were adult patients (> 18 years old) who had an APC1 or APC2 injury [4] based on assessment of the preoperative static radiographs and who consequently underwent EUA. Exclusion criteria were patients with an apparent APC3 (i.e. anterior and posterior SI joint diastasis) or vertical shear injury as diagnosed on the preoperative radiographs and CT scan, and therefore not requiring EUA. The sample size was determined by the availability of eligible participants within the time frame of the study (2017–2024). In patients with pelvic ring injuries, EUA is performed as standard care. For patients with potential hemodynamic instability, CT scans were performed with pelvic binders on. All patients were informed about the study and the EUA procedure, and their consent was documented in Roqua, an electronic research platform used for collecting and managing study-related data. In compliance with privacy regulations, all patient data were pseudoanonymized. The local Medical Ethical Review Board reviewed the methods employed and approved the study (METc 2017/543).

#### Examination under anaesthesia

EUA was performed in the operating room by two pelvic trauma surgeons, both with at least 10 years of experience in operating on pelvic injuries. A radio-opaque, metric ruler was positioned on the skin over the pelvis during each fluoroscopic image to correct for magnification errors. The test procedure was first performed according to the technique described by Sagi et al. [7]. Our study expands on the previously described EUA protocol by introducing additional imaging of the posterior pelvic ring, specifically of the SI joints using obturator inlet projections.

Observation of the anterior pelvic ring included five fluoroscopic images (focusing on the symphysis) taken during static, internal rotation stress, external rotation stress, and longitudinal push/pull stress manoeuvres, in three different fluoroscopic projections: anteroposterior (AP), inlet, and outlet [7]. This process resulted in a total of 15 distinct images for each patient.

**Fig. 2:**
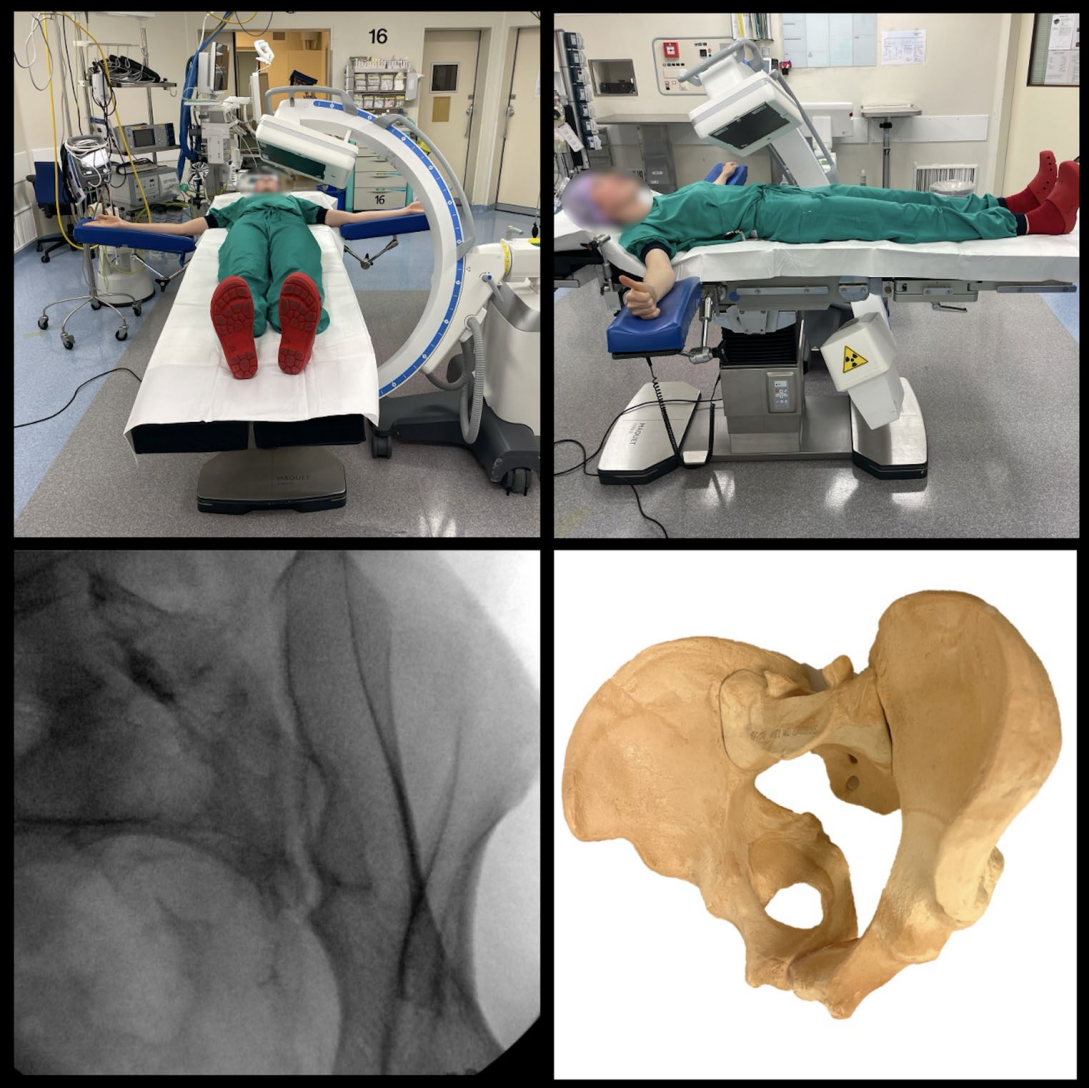
Obturator inlet projection

For the additional observation of the posterior pelvic ring, stress manoeuvres, including static, internal rotation stress, external rotation stress, were performed in projections with an obturator inlet view of both the left and right SI joint as demonstrated in Fig. 2. This allowed direct observation of diastasis of the anterior and posterior SI joints during the various stress manoeuvres [20–22]. This process resulted in a total of 6 additional images (i.e. 3 images of the left SI joint and 3 of the right SI joint) for each patient. Sectra UniView Picture Archiving and Communication System (PACS) was used to observe all images.

### Data collection

Data was prospectively collected from patients’ electronic files. Information regarding age, gender, injury mechanism, measurements of symphyseal diastasis visible on CT scans, classification based on the CT scans, and the use of a pelvic binder during CT scans was recorded. Preoperative CT scans and intraoperative fluoroscopic images from the EUA were reassessed collaboratively by two consultant pelvic trauma surgeons and a pelvic trauma research fellow, discussing the findings until consensus was reached. Symphyseal diastasis was measured on the axial CT scans by locating the narrowest point of the symphysis. All pelvic CT images were calibrated, and metric validation was verified by our radiology department to ensure measurement accuracy. Symphyseal diastasis was first measured in millimetres and then presented in centimetres. All injuries were initially classified using the preoperative CT scans, based on the apparent diastasis in the SI joint. Injuries with no evidence of SI ligament disruptions were classified as APC1, injuries with anterior SI diastasis as APC2, and injuries with anterior and posterior SI diastasis as APC3.

**Fig. 3:**
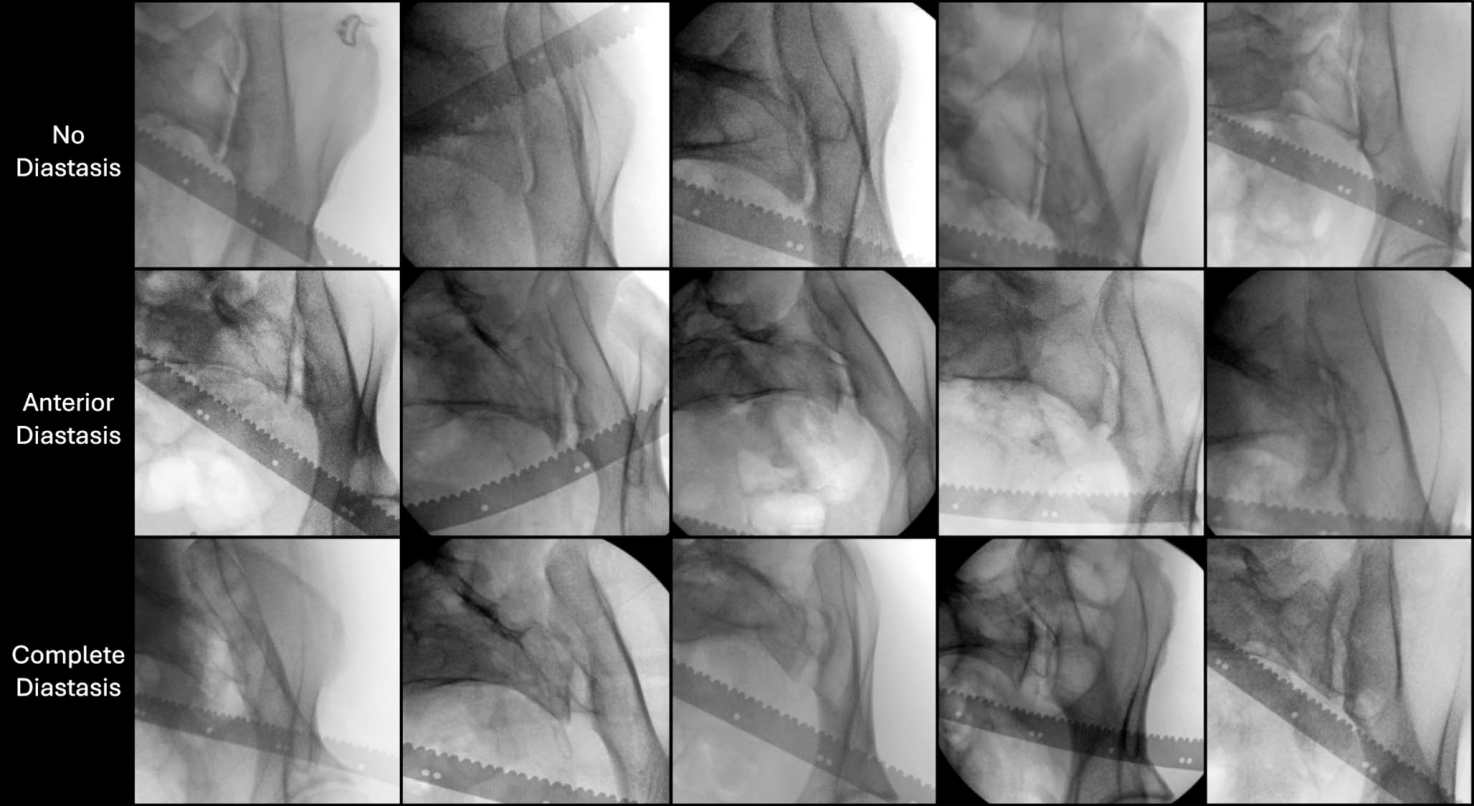
Examples of radiographs of the sacroiliac (SI) joints taken in the external rotation stress maneuver from the obturator inlet projection, with either no diastasis (row 1), anterior diastasis (row 2) or complete diastasis (row 3). As seen in the figure, patients with no SI diastasis had little to no displacement of the ilium, patients with anterior SI diastasis had an ilium that pivoted laterally around the posterior SI joint, indicating intact posterior ligaments, and patients with complete SI diastasis presented with a laterally displaced ilium

Displacement in the anterior pelvic ring during EUA was measured in centimetres using the radio-opaque metric ruler. Measurements included the absolute values of the symphyseal diastasis, and the displacement of the pubic body in the sagittal plane (rotational instability), as described by Sagi et al. [7]. Also, information obtained from each obturator inlet projection regarding the SI joint diastasis was recorded in terms of: no diastasis, anterior diastasis or complete diastasis (anterior and posterior). The post-EUA classification was based on the observations of the SI joint from the obturator inlet projections: APC1 was defined as no diastasis, APC2 as anterior diastasis, and APC3 as anterior and posterior diastasis. All three radiographs of each SI joint were used to determine the amount of SI diastasis as a result of the stress manoeuvres, which helped group patients into the three abovementioned groups. The most severe of the two SI joints determined the classification of each patient. See Fig. 3 for examples of each category.

### Statistical analysis

Descriptive statistics were performed to present patient characteristics. Normally distributed data are presented as means with standard deviations, while non-normally distributed data are reported as medians with interquartile ranges. Categorical variables are presented as frequencies and percentages. All statistical analyses were conducted using SPSS version 28. Results with a p-value ≤ 0.05 were considered statistically significant.

The numbers of patients reclassified as a result of the modified EUA protocol were analysed. Groups were made of patients who were reclassified from APC1 on the CT scans to APC2 or APC3 after the modified EUA protocol, and from APC2 on the CT scans to APC3 after the modified EUA protocol.

Furthermore, situations were identified where the direct observation of the SI joint during EUA allowed localisation of SI diastasis, which was not otherwise observed on CT scans. For instance, in cases where CT images showed no apparent SI diastasis, yet EUA images revealed unilateral or bilateral SI diastasis, or where CT suggested unilateral SI diastasis, but EUA images confirmed it to be bilateral.

The mean symphyseal diastasis from both the CT images and the EUA images was presented, along with the frequency of SI joints with no diastasis, anterior diastasis, and complete diastasis were analysed for both the CT images and the modified EUA protocol images.

The difference in classification following the modified EUA protocol compared to static imaging was assessed using a Chi-square test, and the difference in the mean symphyseal diastasis on EUA images compared to CT was assessed using T-tests, presented with statistical significance and the 95% confidence interval (CI). Furthermore, the association between symphysial diastasis on EUA images and classification based on the modified EUA protocol was tested using a one-way ANOVA test.

To determine the difference in observing the SI joints using the modified EUA protocol compared to the EUA method which only observes the displacement of the anterior pelvis, the cut-off values for anterior pelvic displacement, as indicated by Sagi et al., were used. The cut-off value of 2.5 cm for symphyseal diastasis assumes the absence of anterior SI joint involvement below this threshold and the cut-off value of 1.0 cm for rotational instability assumes no posterior SI joint diastasis below this threshold. Patients who did not meet these cut-off values, yet did have diastasis in the SI joint, were identified. Bar graphs were made to visualize patients in each group with displacement less than and greater than the cut-off values. The difference in classification following the modified EUA protocol compared to the method of only observing the anterior pelvic ring was assessed using a Chi-square test.

## Results

### Study population

A total of 35 patients met the inclusion criteria. Thirty-two patients with an APC3 and 50 patients with a vertical sheer injury based on static images were excluded. The mean age was 53 years (SD 17), with 34 (97.1%) males. The trauma mechanisms included: 2 (5.7%) pedestrian vs. car, 11 (31.4%) falls, 10 (28.6%) motorbike accidents, 4 (11.4%) horse-related accidents, 4 (11.4%) car accidents, 1 (2.9%) bicycle accident and 3 (8.6%) compression/crush injuries. Twenty-eight (80%) were high-energy traumas, meaning that the speed of impact was higher than 20 km/hour or that the fall was from more than two times the height of the patient. Six patients (17.1%) sustained additional lower extremity injuries.

### Preoperative CT scan versus EUA of the anterior and posterior pelvic ring

Based on the initial static CT scans, 12 (34.3%) patients were originally classified as APC1 and 23 (65.7%) as APC2. A total of 23 (65.7%) of patients had an injury that was reclassified following the modified EUA protocol. Accordingly, nine (75%) of the 12 initially classified APC1 injuries were re-classified as APC2 and 1 (8.3%) as APC3. Of the 23 patients initially classified as APC2 injuries, 13 (56.5%) were re-classified as APC3. The difference in classification based on the modified EUA protocol compared to static imaging was statistically significant (*p* = 0.007). Pre- and post-EUA classifications can be seen in Table 1 for each patient, and an overview of reclassifications can be seen in Table 2. Eleven (31.4%) patients had concomitant fractures of the anterior pelvic ring.

**Table 1:**
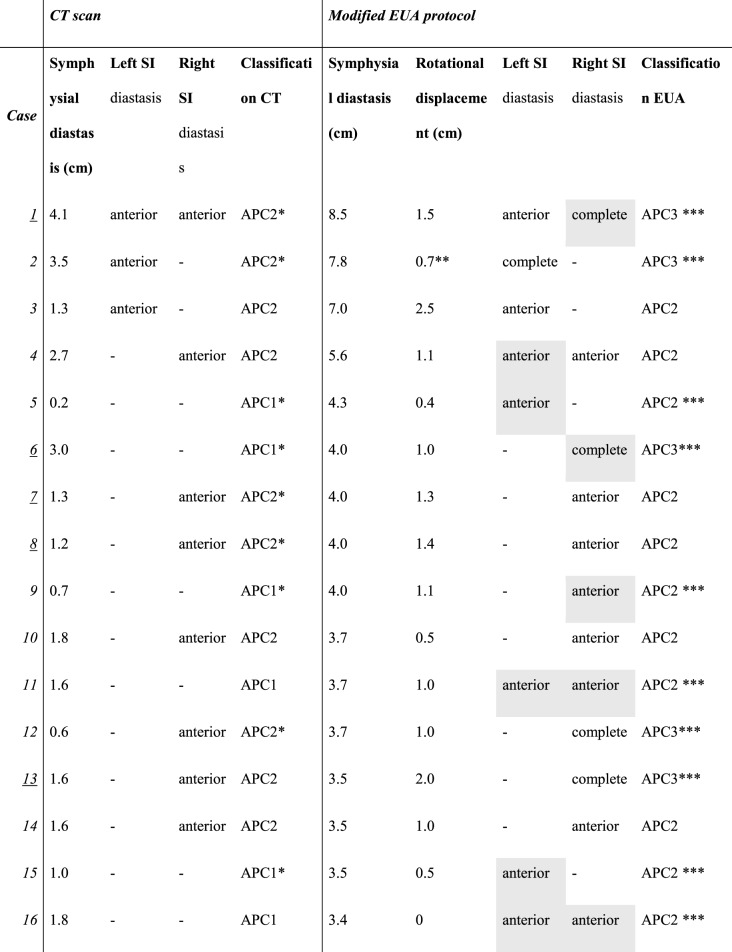

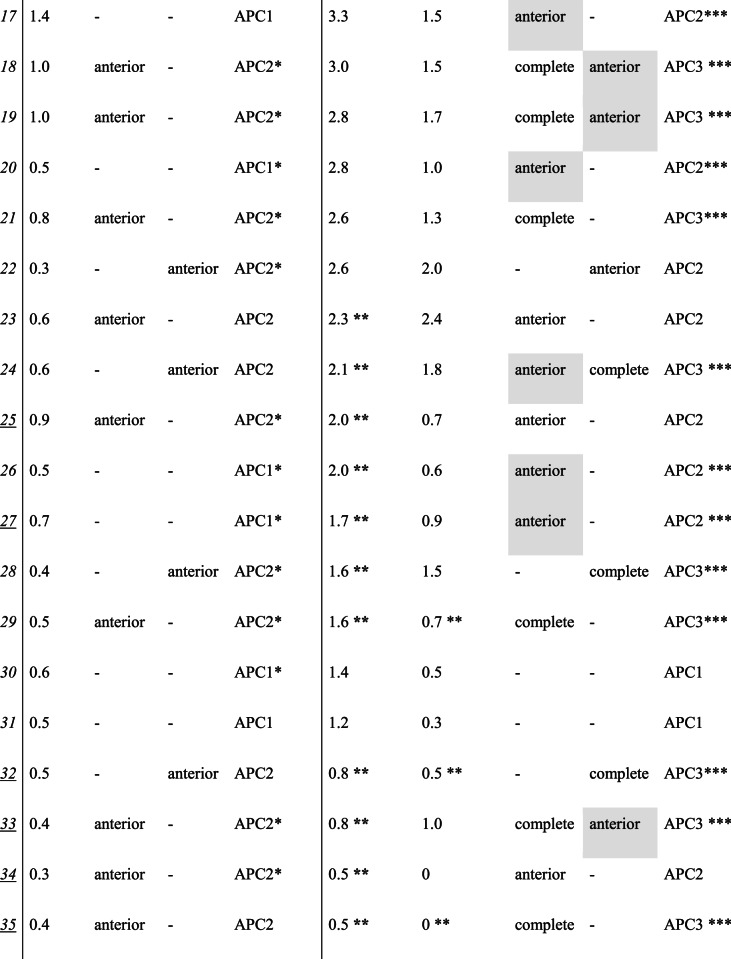
Computerized tomography (CT) scan and examination under anesthesia (EUA) results from the modified protocol

* Use of a pelvic binder during computerized tomography scans **Anterior-posterior compression 2 (APC2) patients with a symphyseal diastasis <2.5cm or anterior-posterior compression 3 (APC3) patients with a rotational displacement of <1.0cm *** Change of classification after examination under anesthesia Situations where observing the SI joint provided important information regarding which SI joint were highlighted in grey The case numbers of patients with contaminant anterior pelvic ring fractures are underlined

**Table 2:** Initially classified anterior posterior compression (APC) 1 and 2 patients based on preoperative computerized tomography (CT) scan scans and the reclassifications following examination under anesthesia (EUA) images from the modified protocol

CT scan	Modified EUA protocol		
	APC1	APC2	APC3
***APC1*** (*n* = 12)	2 (17%)	9 (75%)	1 (8%)
***APC2*** (*n* = 23)	0	10 (43%)	13 (57%)

Table 3 presents the mean symphyseal diastasis measured on the CT scans and on the EUA images, for patients initially classified as APC1 or APC2, along with the frequency of no diastasis, anterior diastasis, and posterior diastasis visible on CT scans and EUA images for all SI joints, taking both the left and right SI joints of each patient into account. The difference of symphysial diastasis on EUA images compared to CT scans was statistically significant (p = < 0.001, 95%CI = 8.2–14.6). There was no significant association between symphyseal diastasis on EUA images and classification (APC1, APC2 or APC3)(*p* = 0.45).

**Table 3:** Diastasis visible on computerized tomography (CT) scan scans vs. examination under anesthesia (EUA) images from the modified protocol

	*Initially classified APC1 injuries* (*n* = 12)		*Initially classified APC2 injuries* (*n* = 23)	
	CT scan	Modified EUA protocol	CT scan	Modified EUA protocol
***Symphyseal diastasis (cm)***,*** mean (SD)***	1.0 (0.8)	2.9 (1.1)	1.2 (1.0)	3.2 (2.2)
***SI joints with no SI diastasis***, n (%)*	24 (100%)	12 (50%)	22 (48%)	17 (37%)
***SI joints with anterior SI diastasis***, n (%)*	0	11(46%)	24 (52%)	17 (37%)
***SI joints with complete SI diastasis***, n (%)*	0	7 (29%)	0	12 (26%)

*Both sacroiliac (SI) joints from each patient were taken into account, meaning there were 24 SI joints for the originally classified APC1 injuries and 46 SI joints for the originally classified APC2 injuries

**Fig. 4:**
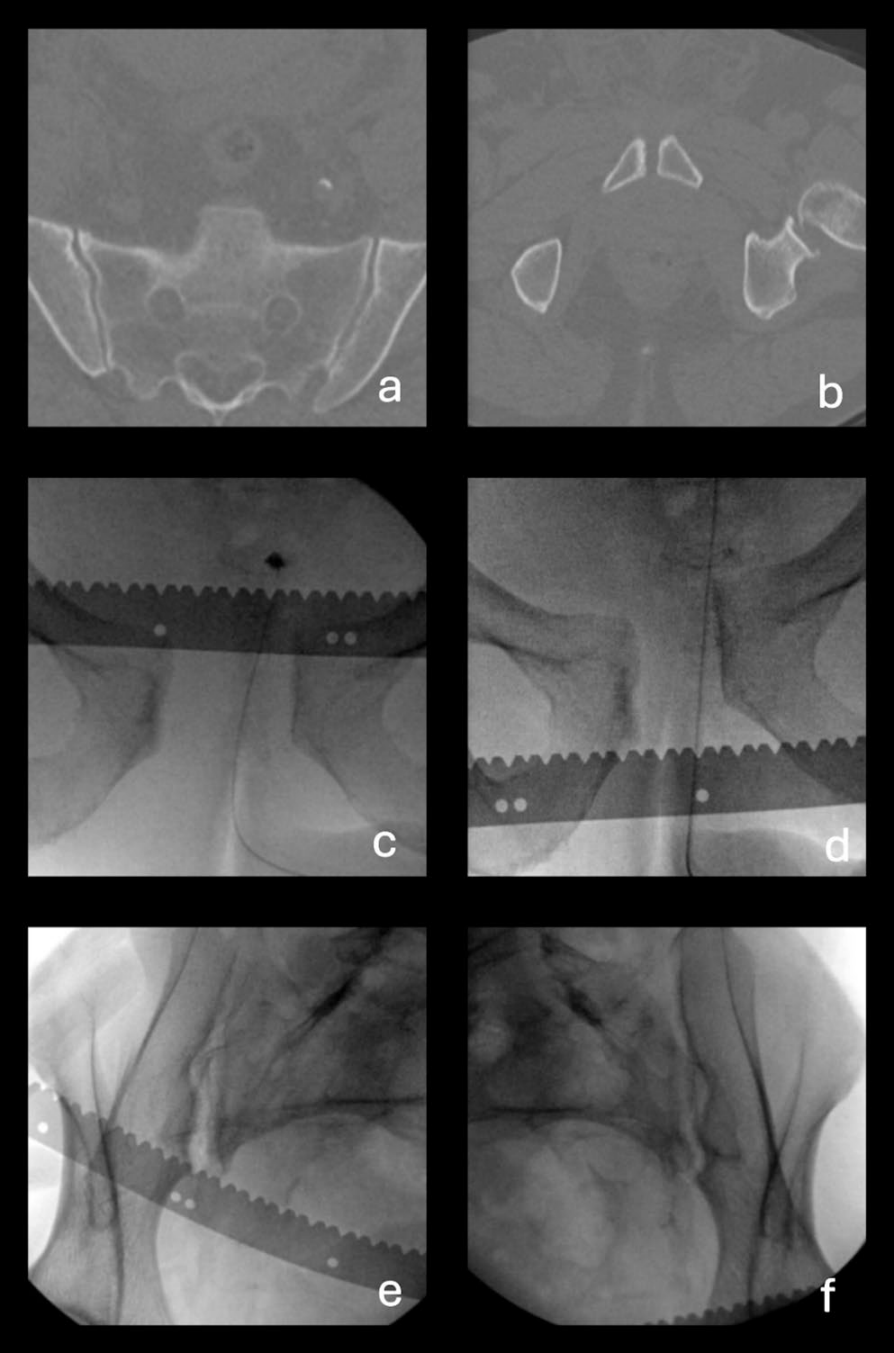
Case 9, which is an example of the classification changing from anterior-posterior compression 1 (APC1) to anterior-posterior compression 2 (APC2), with the added value of observing the sacroiliac (SI) joint being the ability to determine which side the diastasis is located on. (**a**) axial CT scan of the sacrum with a pelvic binder on, showing no apparent SI diastasis (**b**) axial CT image of pubic symphysis showing minimal symphyseal diastasis (**c**) Anterior-posterior (AP) examination under anaesthesia (EUA) image of pubic symphysis in the external rotation stress maneuver, showing 4 cm of diastasis, but providing no insight into which SI joint is affected (**d**) AP EUA image of pubic symphysis in the push/pull stress maneuver, showing 1.1 cm of rotational instability, but providing no insight into which SI joint is affected (**e**) Obturator inlet EUA image of right SI in the external rotation stress maneuver, demonstrating diastasis in the anterior SI joint (**f**) Obturator inlet EUA image of left SI in the external rotation stress maneuver, showing no SI diastasis

**Fig. 5:**
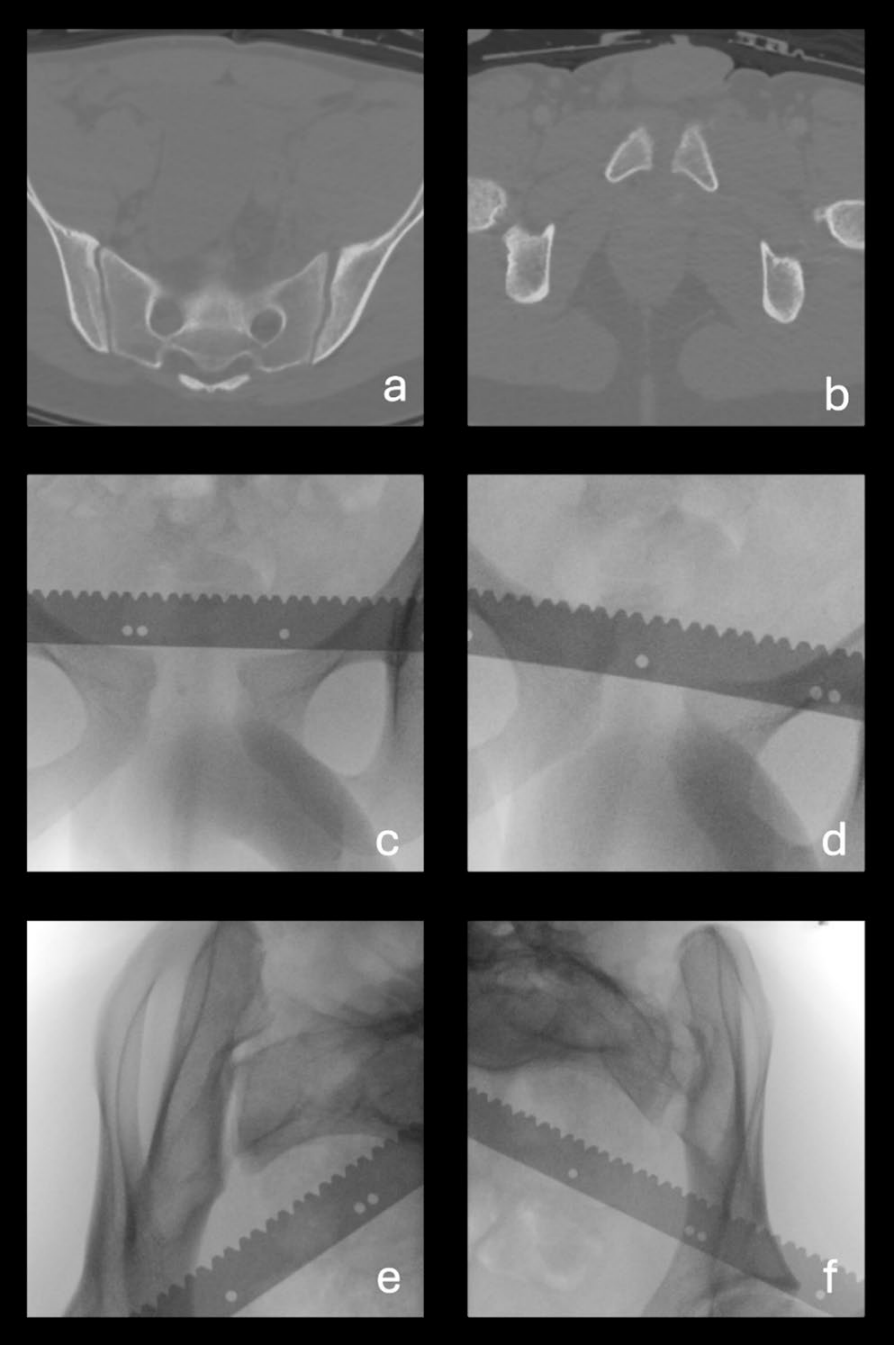
Case 18 is an example of when the classification changes from anterior-posterior compression 2 (APC2) to anterior-posterior compression 3 (APC3), where observing the sacroiliac (SI) joint allowed us to detect diastasis in both SI joints, instead of assuming that there is only diastasis in the left SI, as seen on the CT scans. (**a**) axial CT image of sacrum with a pelvic binder on, demonstrating diastasis in the anterior part of the left SI joint but no apparent diastasis in the posterior part of the SI joint (**b**) axial CT image of pubic symphysis, showing minimal symphyseal diastasis (**c**) Anterior-posterior (AP) examination under anaesthesia (EUA) image of the pubic symphysis in the external rotation stress maneuver showing 3.0 cm of symphyseal diastasis (**d**) AP EUA image of the pubic symphysis in the push/pull stress maneuver showing 1.5 cm of rotational instability (**e**) Obturator inlet EUA image of right SI in the external rotation stress maneuver, demonstrating anterior SI diastasis (**f**) Obturator inlet EUA image of left SI in the external rotation stress maneuver, demonstrating complete SI diastasis

In 16 cases (45.7%), examining the SI joint using the modified EUA protocol revealed important information about which SI joint was affected. These are highlighted in grey in Table 1, and examples of these cases are further illustrated in Figs. 4 and 5.

### EUA of only the anterior pelvic ring versus EUA of the anterior and posterior pelvic ring

**Fig. 6:**
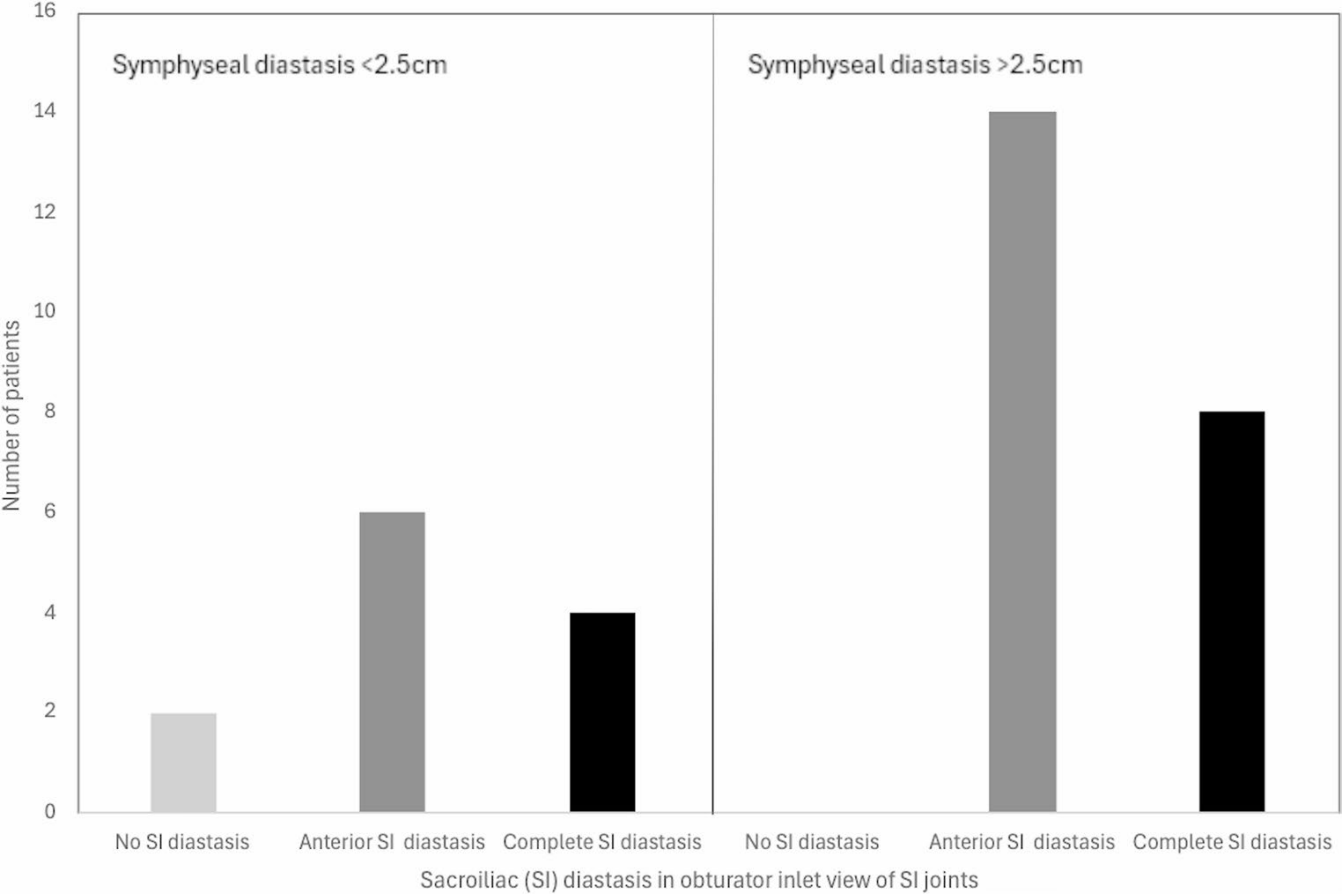
Sacroiliac (SI) diastasis observed during examination under anaesthesia in obturator inlet view of the sacroiliac joints in patients with a symphyseal diastasis of < 2.5 cm and > 2.5 cm. This shows that SI diastasis can occur in patients with minimal diastasis of the pubic symphysis

Eleven out of 13 patients with a symphyseal diastasis of less than 2.5 cm, as measured during EUA on imaging of the anterior pelvic ring, turned out to have occult SI diastasis that was visible on the obturator inlet projections of the SI joints. Five of these patients had anterior SI joint diastasis (APC2), and 6 patients had complete SI diastasis (APC3). This is presented in Fig. 6.

**Fig. 7:**
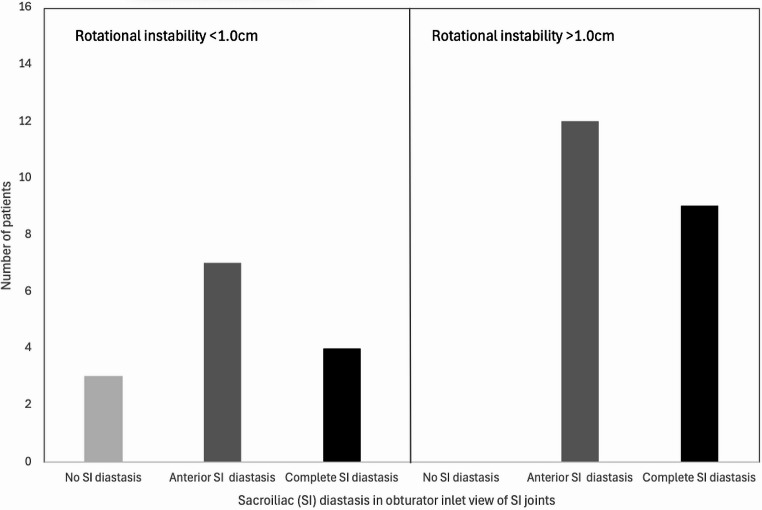
Sacroiliac (SI) diastasis observed in obturator inlet view of the sacroiliac joints in patients with a rotational instability of < 1.0 cm and > 1.0 cm. This shows that complete SI diastasis can occur in patients with minimal rotational instability of the anterior pelvic ring

**Fig. 8:**
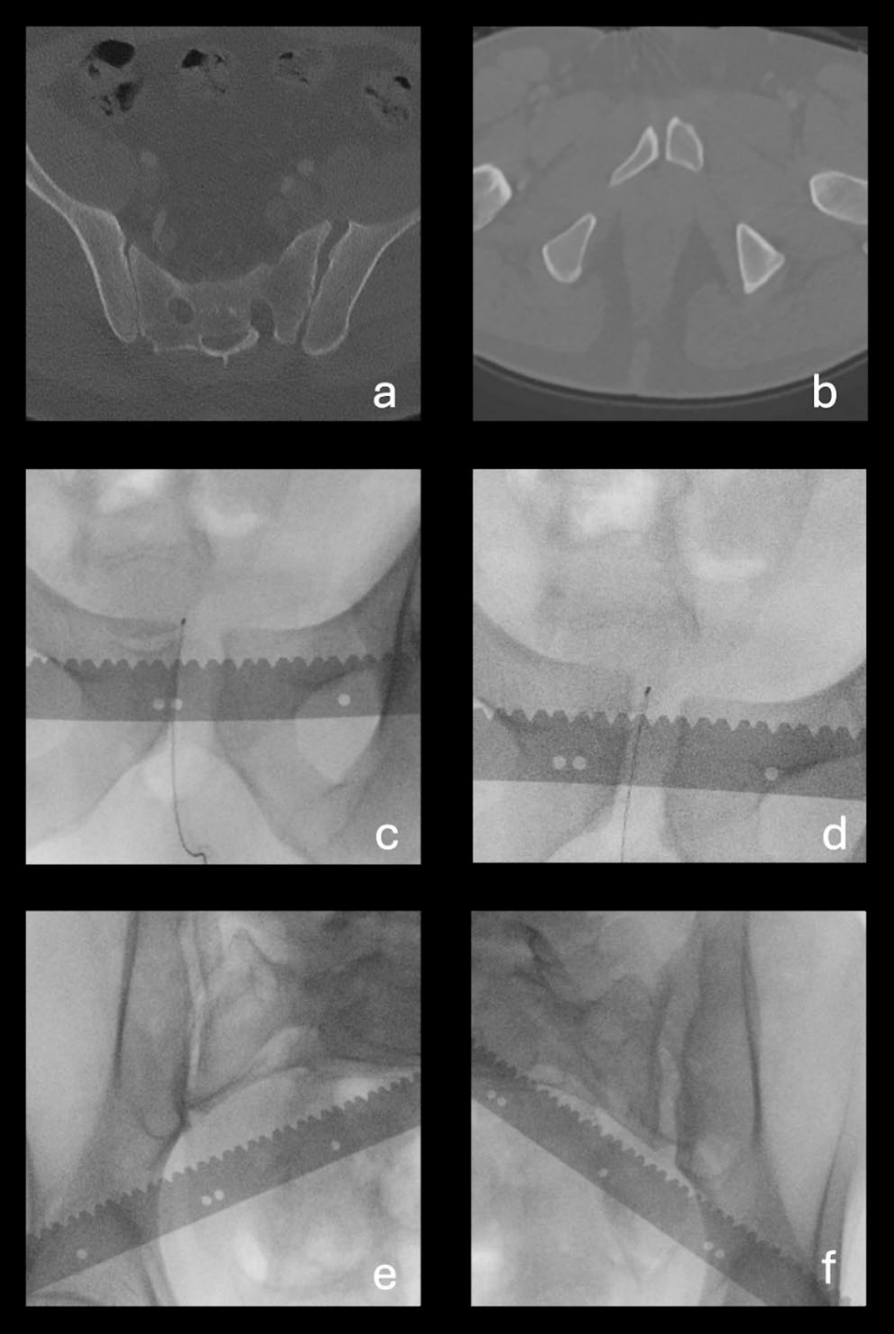
Case 29 is an example of a case when there is substantial sacroiliac (SI) joint diastasis, which is not reflected by the measurements of displacement in the anterior pelvis. (**a**) axial CT scan of the sacrum with pelvic binder on, showing diastasis in the left SI joint (**b**) axial CT image of pubic symphysis showing minimal symphyseal diastasis (**c**) anterior-posterior (AP) examination under anaesthesia (EUA) image of the pubic symphysis in the external rotation stress maneuver, showing 1.6 cm of symphyseal diastasis (**d**) AP EUA image of the pubic symphysis in the push/pull stress maneuver, showing 0.7 cm of rotational instability (**e**) Obturator inlet EUA image of right SI joint in the external rotation stress maneuver, demonstrating no SI diastasis (**f**) Obturator inlet EUA image of left SI joint in the external rotation stress maneuver, showing complete SI diastasis

**Fig. 9:**
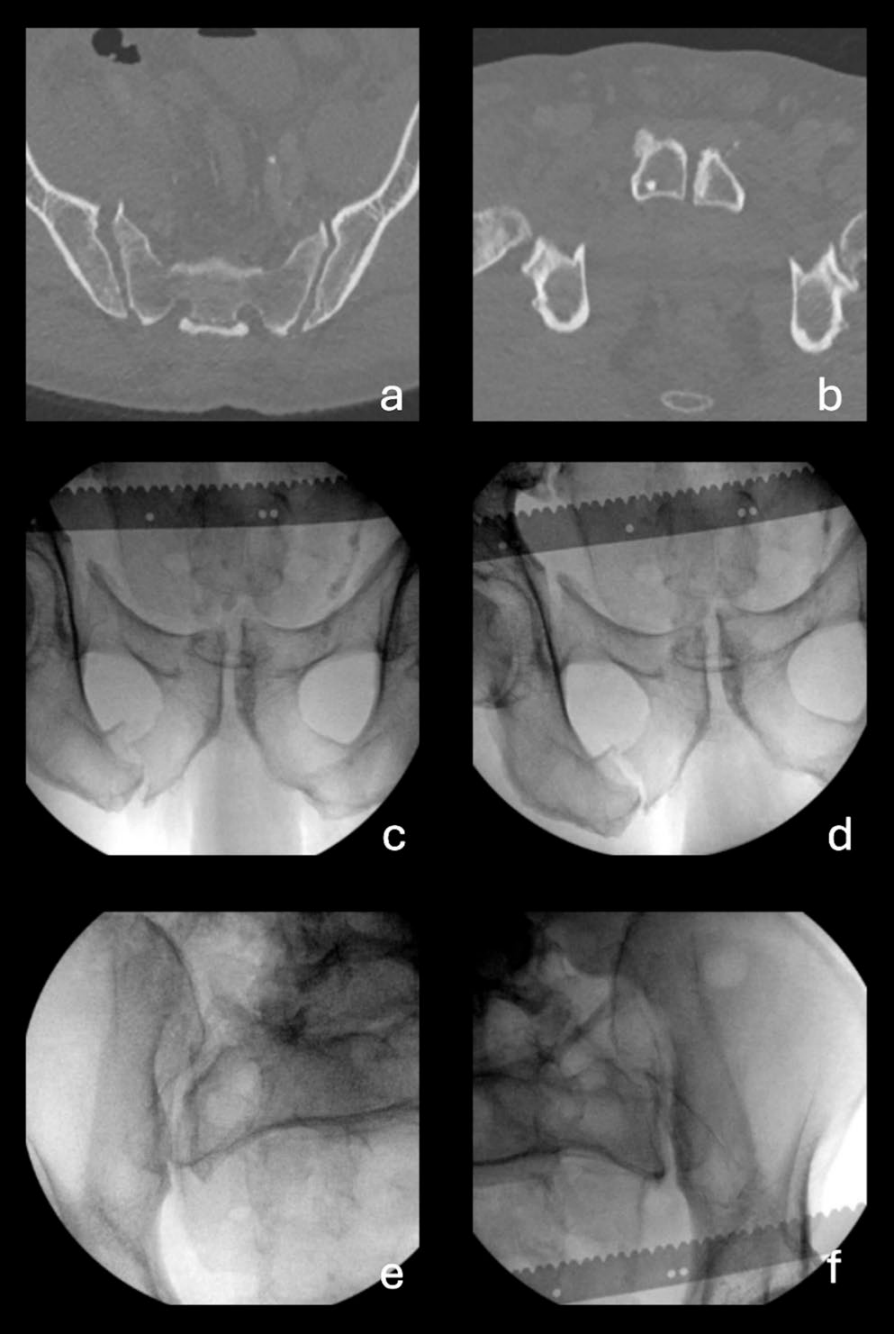
Case 32 is an example of a case when there is substantial sacroiliac (SI) joint diastasis, which is not reflected by the measurements of displacement in the anterior pelvis, potentially associated with fractures of the superior and inferior pubic rami (**a**) axial CT scan of the sacrum without pelvic binder on, showing diastasis in the right SI joint (**b**) axial CT image of pubic symphysis showing minimal symphyseal diastasis (**c**) anterior-posterior (AP) examination under anaesthesia (EUA) image of the pubic symphysis in the external rotation stress maneuver, showing 0.8 cm of symphyseal diastasis (**d**) AP EUA image of the pubic symphysis in the push/pull stress maneuver, showing 0.5 cm of rotational instability (**e**) Obturator inlet EUA image of right SI joint in the external rotation stress maneuver, demonstrating complete SI diastasis (**f**) Obturator inlet EUA image of left SI joint in the external rotation stress maneuver, showing no SI diastasis

Four patients out of 14 with a rotational instability of less than 1.0 cm, as measured during EUA on imaging of the anterior pelvic ring, turned out to have complete diastasis (APC3) visible on the obturator inlet projections of the SI joints. These cases are depicted in Fig. 7. See Figs. 8 and 9 for examples of cases where the anterior pelvic displacement is minimal, but substantial SI joint diastasis can be observed.

The overall difference in classification based on the modified EUA protocol compared to the method only observing the anterior pelvic ring was not statistically significant (*p* = 0.259).

## Discussion

Our modified EUA protocol resulted in a reclassification of 66% of injuries in this patient group. Furthermore, in almost half of the patients, the modified EUA protocol allowed us to determine if the diastasis was located in the left, right or both SI joints. This information could not have been obtained through the use of CT images or the conventional EUA protocol. Lastly, in 34% of patients, the presence of diastasis in the SI joint was observed that would have otherwise been missed if only the theoretical instability of the SI joints based on the standard cut-off values of the anterior pelvic displacement had been assessed. Therefore, the addition of the obturator inlet projections of the SI joints is a valuable addition to the EUA procedure for APC injuries.

The first research question focused on the added value of the modified EUA protocol compared to static radiographs or CT images. Several articles have demonstrated that the use of EUA is effective in identifying occult instability in patients with pelvic ring injuries, compared to using static imaging only [6, 7, 9, 16]. Suzuki et al. observed that the mean symphyseal diastasis increased from 1.4 cm on CT scans to 2.5 cm after EUA in patients initially assumed APC1 injuries [6]. Similarly, our study showed an increase in symphyseal diastasis from a mean of 1.0 cm on CT scans to 2.9 cm after EUA for patients who were initially classified as APC1. In patients initially classified as APC2, the symphyseal diastasis increased from 1.2 cm to 3.2 respectively, in our study. Furthermore, Sagi et al. showed that 50% of APC1 injuries and 39% of APC2 injuries were reclassified after performing EUA; however, their study did not provide information regarding which side the SI joint was affected, as the posterior pelvic ring was not assessed [7]. There has not yet been a study that directly described the SI joint during EUA.

The second research question focused on the added value of observing the SI joints using the modified EUA protocol compared to the traditional EUA method, which only observes the displacement of the anterior pelvic ring. Several studies support the necessity of observing both the anterior and posterior pelvic rings, rather than relying solely on the anterior pelvic ring and the established cut-off values.

The cut-off value of 2.5 cm was initially reported by Pennal et al. [3] and Tile [2], stating that a symphyseal diastasis of more than 2.5 cm is associated with a disruption of the anterior SI ligaments. This finding was based on biomechanical studies on cadavers; however, they did not describe their study population or methods of measurement, and there is little further evidence supporting this value [6, 23].

In a recent biomechanical study, Doro et al. investigated the 2.5 cm cut-off value, but were unable to confirm its validity in distinguishing the presence of anterior SI ligament disruption [17]. Their study of 20 cadavers reported that the symphyseal diastasis ranged from 1.8 cm to 4.4 cm before anterior SI ligaments failed [17]. This variability aligns with our results and supports the need for directly assessing the SI joint during EUA.

Although Whiting et al. reported a 100% negative predictive value of EUA using the 2.5 cm cut-off value, only 8 patients with APC1 injuries were included, and the only outcomes used were the occurrence of a delayed operative fixation and a measurement of symphysis diastasis on an AP radiograph during follow-up, which was not decreased in all patients [24]. Furthermore, Young et al. suggested that the presence of a pubic ramus or acetabulum fracture may be associated with a reduced threshold for the diastasis in the symphysis before affecting the SI ligaments [4], which is also reflected in our results (Fig. 9). Patients with APC injuries that are not purely ligamentous have not been taken into consideration in the traditional EUA procedure. Observing the SI joint in patients with fractures allows detection of occult SI joint diastasis in this patient group as well, as the fractures may result in unpredictable amounts of diastasis in the posterior pelvic ring.

Preoperative MRI studies have also highlighted limitations of the anterior-only assessment. Rojas et al. [25] reported two patients with SI joint injuries on MRI whose symphyseal diastasis during EUA measured less than 2.5 cm. Both patients, treated according to the EUA protocol suggested by Sagi et al., experienced severe pain and instability on follow-up radiographs, requiring delayed surgical fixation. These findings align with our results and those of Doro et al. [17], demonstrating that SI ligament disruption can occur even when symphyseal diastasis is below 2.5 cm.

Moreover, there is no scientific literature supporting the cut-off value of 1.0 cm for rotational instability [7]. Consequently, the extent of SI injury and treatment cannot be determined solely based on the displacement of the anterior pelvic ring, as diastasis may be missed, and patients may not receive the appropriate treatment for their injury. This is clinically relevant as misclassification and inadequate treatment may result in poor functional outcomes, malunion and re-operation [6, 7, 12–15, 26, 27].

Although this study presents a method for identifying initial SI diastasis, the correlation between pelvic diastasis, initial or residual, and clinical outcomes remains a subject of debate. A common criticism of existing literature advocating for treatment based solely on radiographic parameters is the lack of patient-reported outcomes at follow-up. Some studies have focused on *residual* (post-reduction) SI joint diastases rather than *initial* (EUA) diastases and their relationship with clinical outcomes, making direct comparisons to our EUA method challenging. Several studies suggest that there is no clear relationship between residual posterior ring diastasis and clinical outcomes [28, 29], while others propose that an anatomic reduction leads to significantly better outcomes [30–35]. However, it is important to note that EUA was not used in these studies to determine initial diastasis. Also, initial dynamic instability in the EUA images does not necessarily mean it will displace to that degree over time. It has, however, been demonstrated in two cases from the study by Rojas et al. [25], as mentioned above, that non-operative treatment in patients with anterior SI diastasis can result in long-term pain and, eventually, delayed surgical intervention. However, presenting an EUA protocol for assessing the *anterior and posterior* pelvic ring, along with a better understanding of these injuries, as shown in this study, seems a crucial first step toward optimizing treatment. Future studies should investigate the relationship between initial SI diastasis observed in EUA images, treatment choices, and outcomes in terms of residual displacement and patient-reported outcomes.

Some strengths and limitations of this study should be addressed. Firstly, all procedures were performed by the same two experienced trauma surgeons, allowing consistency of the procedure and the results. Furthermore, this study included a representative group of APC injuries presented in a level-one trauma centre, allowing findings to be applied to all APC1 and APC2 injuries in other centres. On the other hand, it should be mentioned that the patient population consisted primarily of men, which may limit generalisability and should be considered a limitation. Lastly, the obturator inlet projection allows a clear view into the SI joint without the view of the SI joint being obscured, as seen in the AP projections [20–22]. However, simultaneous comparability of both SI joints is not possible with this method. Furthermore, measurements of the amount of SI joint diastasis cannot be provided with this method. This is because there is no widely accepted distance that indicates a disruption of the ligaments due to morphologic variations of the SI joint [20]. Instead, whether there was no diastasis, anterior SI diastasis, or complete SI diastasis was determined by experienced pelvic trauma surgeons, which may introduce bias. Future studies are warranted to assess the intra-observer reliability of this method. Additionally, the majority of preoperative CT scans were executed when the patients had pelvic binders on. This may result in higher rates of reclassification following EUA compared to CT scans taken without pelvic binders [9]. On the other hand, this was done according to standard clinical practice, making it representative of level-1 trauma centres [34, 35]. Furthermore, patients in this study were not treated according to a specific treatment protocol based on the results of EUA, as in other studies [7, 25]. Such a protocol can only be properly developed and applied safely once a clearer understanding of the injury instability and the EUA method is obtained. Future studies should further examine this modified protocol so that a treatment protocol can be developed. The goal of this study was to determine if initial occult SI diastasis could be detected, and with this information, future studies should be conducted to determine the optimal treatment based on patient outcomes. Lastly, another limitation may be the small sample size, as it limits statistical power and generalizability.

**Fig. 10:**
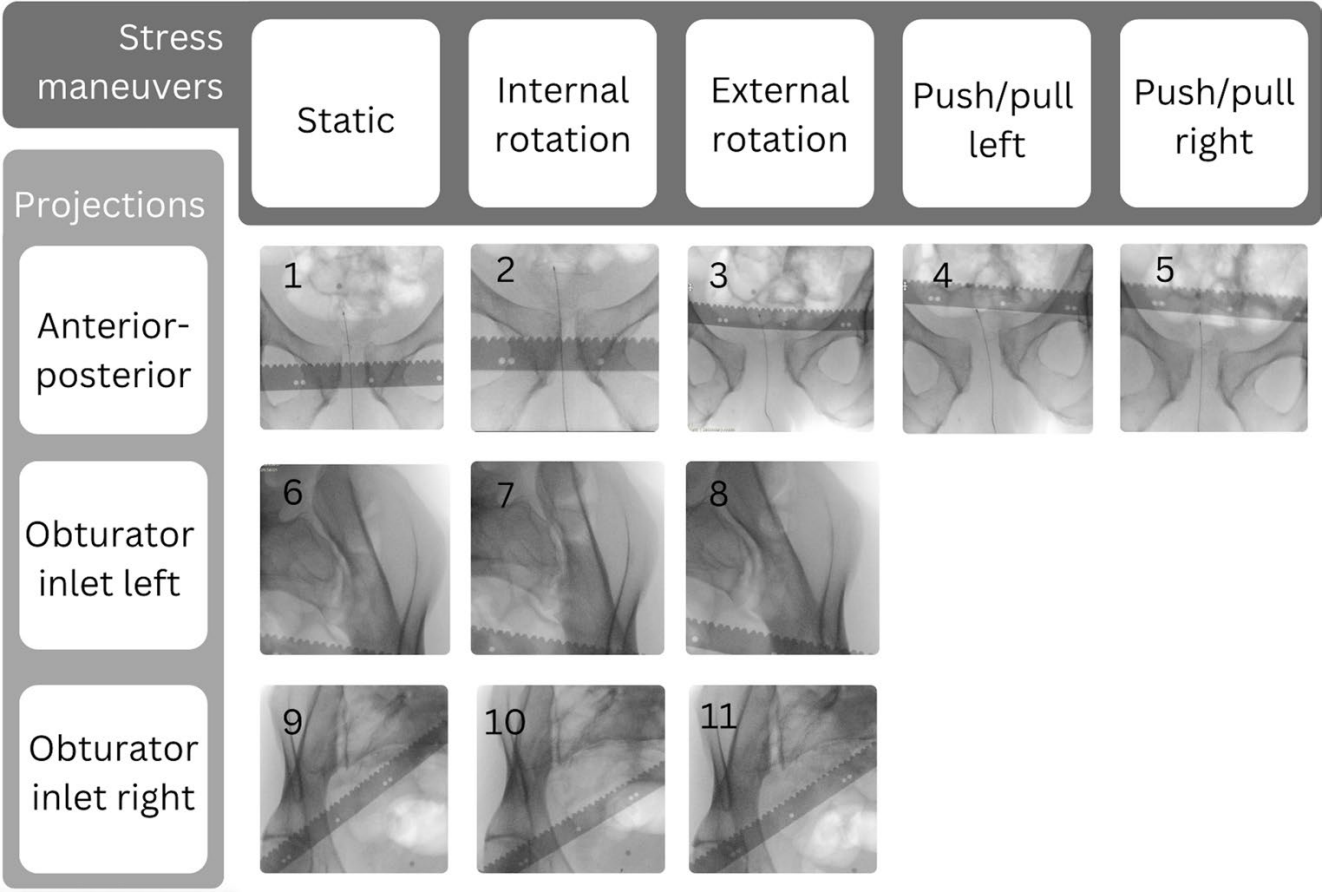
Modified Examination Under Anaesthesia protocol for patients with anterior posterior compression injuries

Based on the findings, a modified EUA procedure for patients with APC injuries, offering the advantage of fewer fluoroscopic images while providing dynamic imaging of both the anterior and posterior pelvic ring, is proposed. This consists of a series of 11, instead of 15 radiographs, including five fluoroscopic images: static, internal rotation stress, external rotation stress, and longitudinal push/pull stress manoeuvres, in the AP projection, and 6 fluoroscopic images: static, internal rotation stress, and external rotation stress from the obturator inlet view of the left and right SI joints, as depicted in Fig. 10. The inlet and outlet views of the anterior pelvic ring did not provide additional diagnostic value, as adequate information regarding the anterior pelvic ring instability can be obtained from the AP images alone. Instead, a focus on the SI joints is added in the modified protocol.

## Conclusions

Observing the sacroiliac joint using obturator inlet projections during EUA in patients with APC injuries is a valuable addition to the protocol, as it allows for direct detection of diastasis that was not visible on the CT scans, allows localization of SI joint diastasis, and reveals SI joint diastasis that would remain undetected if only the anterior ring was visualized. As a result, a modified protocol that minimizes the number of fluoroscopic images, while observing both the anterior and posterior pelvic ring has been proposed. Further research is warranted to validate this technique and determine its utility in informing optimal treatment strategies.

## Data Availability

No datasets were generated or analysed during the current study.
